# Small Interfering RNA Efficiently Suppresses Adhesion Molecule Expression on Pulmonary Microvascular Endothelium

**DOI:** 10.4061/2011/694789

**Published:** 2011-10-05

**Authors:** Tobias Walker, Julian Siegel, Andrea Nolte, Silke Hartmann, Angela Kornberger, Volker Steger, Hans-Peter Wendel

**Affiliations:** ^1^Department of Thoracic, Cardiac and Vascular Surgery, Tübingen University Hospital, Hoppe-Seyler-Straße 3, 72076 Tübingen, Germany; ^2^Division of Congenital and Paediatric Cardiac Surgery, Clinical Research Laboratory, Children's Hospital, Tübingen University Hospital, Hoppe-Seyler-Straße 3, 72076 Tübingen, Germany; ^3^Department of Anaesthesiology and Intensive Care, Tübingen University Hospital, Hoppe-Seyler-Straße 3, 72076 Tübingen, Germany

## Abstract

*Background*. Adhesion molecules are known to influence postoperative organ function, they are hardly involved in the inflammatory response following the ischemia-reperfusion injury. We sought to investigate the potency of small interfering RNAs to suppress adhesion molecule expression in human pulmonary microvascular endothelial cells. *Methods*. Human lung microvascular endothelial cells were transfected with specific siRNA followed by a stimulation of the cells with an inflammatory cytokine. Adhesion molecule expression was determined by FACS-analysis, and reduction of intracellular mRNA was determined by qRT-PCR. Furthermore, the attachment of isolated neutrophils on the endothelial layer was determined after siRNA transfection. *Results*. In summary, siRNA transfection significantly decreased the percentage positive cells in a single cocktail transfection of each adhesion molecule investigated. Adhering neutrophils were diminished as well. *Conclusion*. siRNA might be a promising tool for the effective suppression of adhesion molecule expression on pulmonary microvascular cells, potentially minimizing leukocyte-endothelial depending interactions of a pulmonary allograft.

## 1. Introduction

Formerly used as an ultimate ratio in end-stage pulmonary diseases, lung transplantation has become a commonly accepted therapy with an increasing number of procedures performed annually. Despite the considerable advantages of perioperative medical care, two main problems remain unresolved: rejection of the graft and primary graft failure (PGF) of the allograft defined by a noncardiogenic pulmonary oedema appearing shortly after reperfusion of the transplanted organ. Large clinical trials report an incidence of between 22% and 57% in patients receiving a lung transplant [1, 2]. A central factor involved in the development of PGF seems to be the expression of adhesion molecules on the surface of pulmonary endothelial cells. They represent a group of different glycoproteins and carbohydrates expressed on the surface of a wide variety of cell types, including endothelial cells. By interfering with receptors on the leukocytes, these adhesion molecules allow initial contact between the leukocytes and the vessel wall. Firm adhesion and transendothelial migration follow, resulting in a sequestration of leukocytes at the endothelium and the later infiltration of the interstitial space. The release of proteolytic enzymes and oxygen-free radicals contributes to damage of the alveolar membrane, resulting in noncardiogenic pulmonary oedema. 

Furthermore, some adhesion molecules seem to be involved in allograft rejection and the development of obliterative bronchiolitis. Different investigators have demonstrated a positive correlation between the intensity of adhesion molecule expression on the endothelium of the allograft vasculature and clinical and histological signs of organ rejection [3, 4].

Therefore, suppression of adhesion molecule expression on the vascular endothelium (thereby interrupting the leukocyte-endothelial interaction) seems to be a promising tool to improve the quality of life of allograft recipients. However, nowadays, no commonly accepted therapeutic possibility exists. We selected three different adhesion molecules as targets. E-selectin from the selectins group modulates the initial establishment of contact and causes rolling of the leukocytes along the vascular endothelium. Intercellular adhesion molecule 1 and vascular adhesion molecule belong to the immunoglobulins group and mediate the fixed connection between leukocytes and endothelium. Other groups of investigators have already demonstrated that adhesion molecules play a key role when it comes to recruitment of leukocytes in lungs, thus modulating the subsequent inflammatory reaction [5].

Using small interfering RNA (siRNA) to develop new therapies seems to show promise. Originally identified as an intermediate of the RNA interference pathway in the late 1980s, siRNA has become a serious alternative in medical research and, therefore, therapeutic application. For practical use, the sequence of the targeted gene has to be identified in a gene library. Analogous to the gene sequence, double-stranded siRNA is manufactured by commercial providers and brought in the cytoplasm by transfection. One strand of siRNA becomes part of a ribonucleoprotein complex called RNA-induced silencing complex (RISC). RISC can cleave RNA sequences complementary to the siRNA strand, thereby causing the rapid degradation of the target mRNA. Importantly, and in contrast to longer double-stranded RNAs, siRNAs in most cases do not induce an interferon response leading to a general cytotoxicity [6]. 

The vision of our working group consists of using siRNAs for effecting temporary suppression of endothelial adhesion molecules in pulmonary allografts in order to reduce the early and longer-lasting losses in functionality described above as well as the increase in patient morbidity and mortality resulting therefrom.

The aim of the present study was to evaluate the potency of specific siRNA to block the expression of intercellular adhesion molecule 1 (ICAM1, CD 54), the vascular cell adhesion molecule 1 (VCAM1, CD 106), and E-selectin (CD 62E) in cultured human pulmonary microvasculature cells.

## 2. Results

### 2.1. Adhesion Protein Receptor Expression after TNF-*α* Stimulation

**Figure 1:**
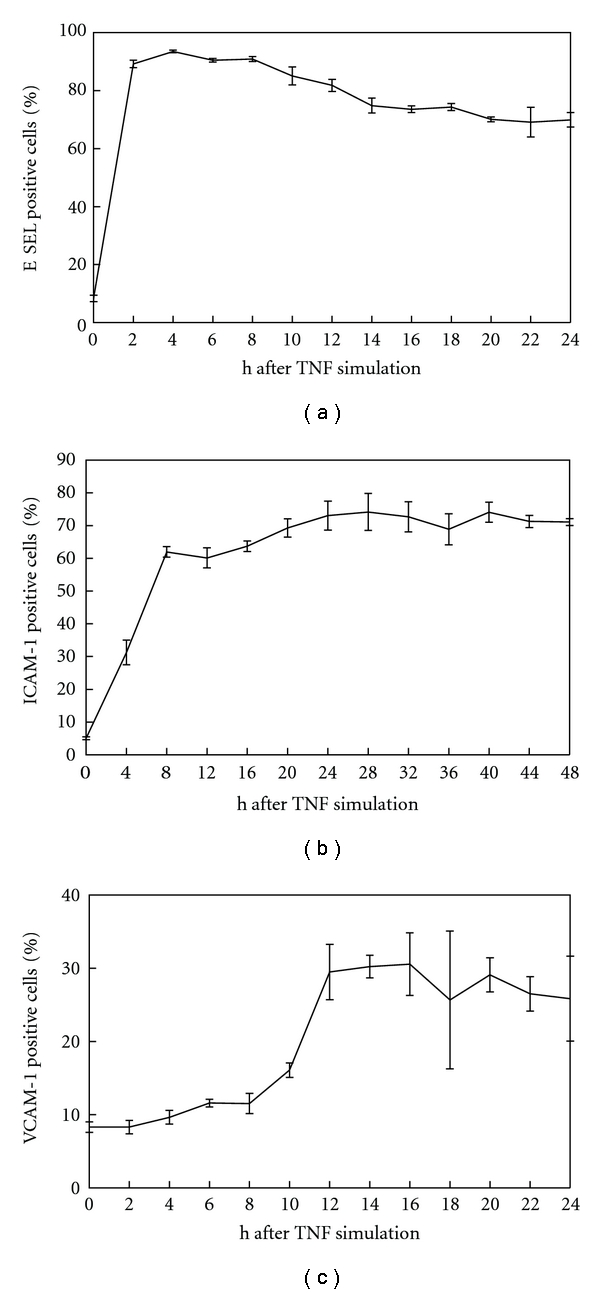
Increase of the adhesion protein receptor expressions of ESELE, ICAM-1, and VCAM-1 during the first 24 hours after TNF-*α* stimulation. The *y*-axis represents the percentage of the respective receptor expression, and the *x*-axis represents the time after TNF-*α* stimulation in hours.

Stimulation of HLMECs with TNF resulted in a significant increase in receptor expression of all three adhesion proteins measured by FACS. Maximal expression was found 4 h and 12 h post-TNF-*α* stimulation for ESELE and VCAM-1, respectively. ICAM-1 expression stayed high between 8 and 48 h (see Figure 1). Concerning all three types of adhesion molecules, the time span of twelve hours after stimulation ensured a high expression level of all adhesion molecules investigated. 

#### 2.1.1. Suppression of Receptor Expression by Single siRNA Transfection

**Figure 2:**
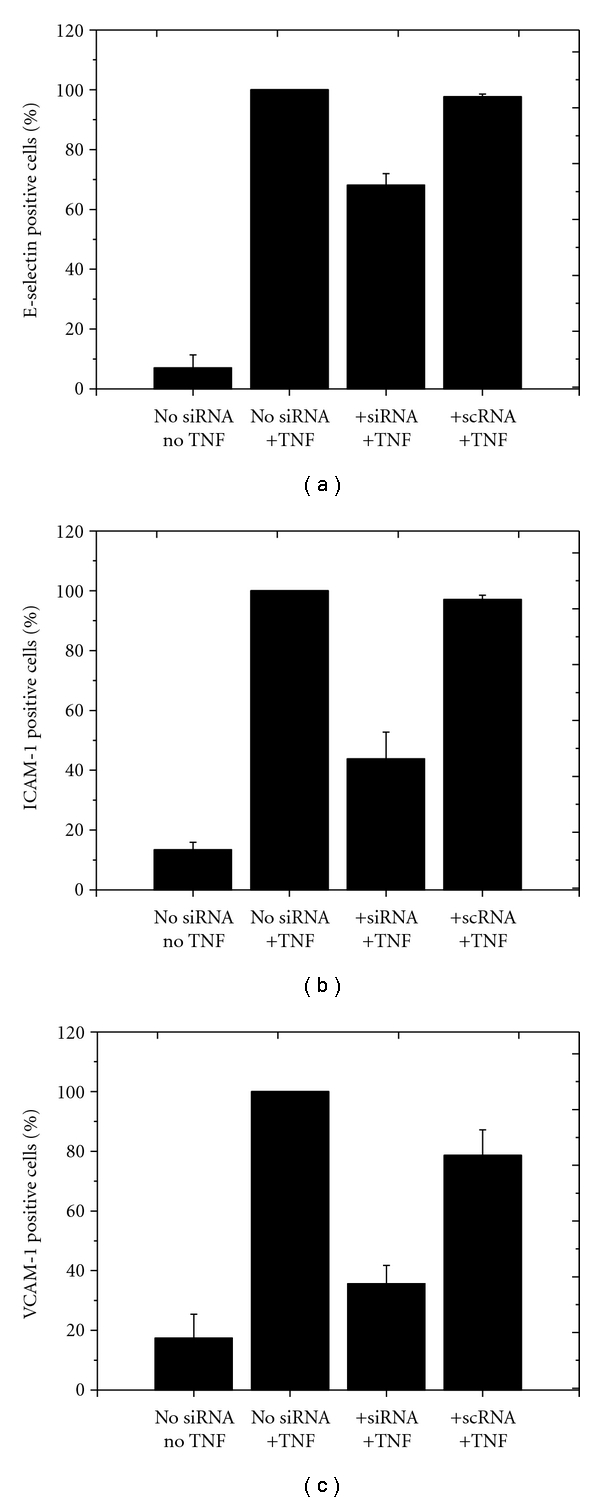
Effects of siRNA and TNF treatment on the fraction of cells positive for ESELE, ICAM-1, and VCAM-1. The *y*-axis reflects the respective adhesion receptor expression (in %), whereby untreated cells stimulated with TNF-*α* (group 2) were set to 100%. *x*-axis: group 1: HLMVECs without siRNA transfection and without TNF-*α* stimulation; group 2: HLMVECs without siRNA transfection, but with TNF-*α* stimulation; group 3: HLMVECs transfected with a receptor-specific siRNA and with TNF-*α* stimulation; group 4: HLMVECs transfected with a nonspecific siRNA and with TNF-*α* stimulation. Each bar represents the mean ± SD of *n* = 3 independent experiments.

FACS analysis showed a significant increase of the expression of all three adhesion proteins after HLMECs stimulation with TNF-*α* (Figure 2). For ESELE expression (Figure 2(a)), the baseline value (no TNF-*α* and no siRNA application) was 7.02 ± 4.36% compared to the TNF-*α* group (100%). Treatment of HLMECs with ESELE specific siRNA resulted in a significant diminished receptor expression of 68.09 ± 3.89% (*P* < .001), whereas the siSCR control group remained at 97.68 ± 0.89%. Application of ICAM-1-specific siRNA led to a significant reduction of the ICAM-1 expression to only 43.80 ± 8.98% (*P* < .001), and treatment of HLMECs with VCAM-1 siRNA (Figure 2(c)) was 35.65 ± 6.11% compared to TNF-*α* stimulated cells (*P* < .001) (see Figure 2).

#### 2.1.2. Suppression of Receptor Expression by Application of an siRNA Cocktail Targeting All Three Different Adhesion Receptors

**Figure 3:**
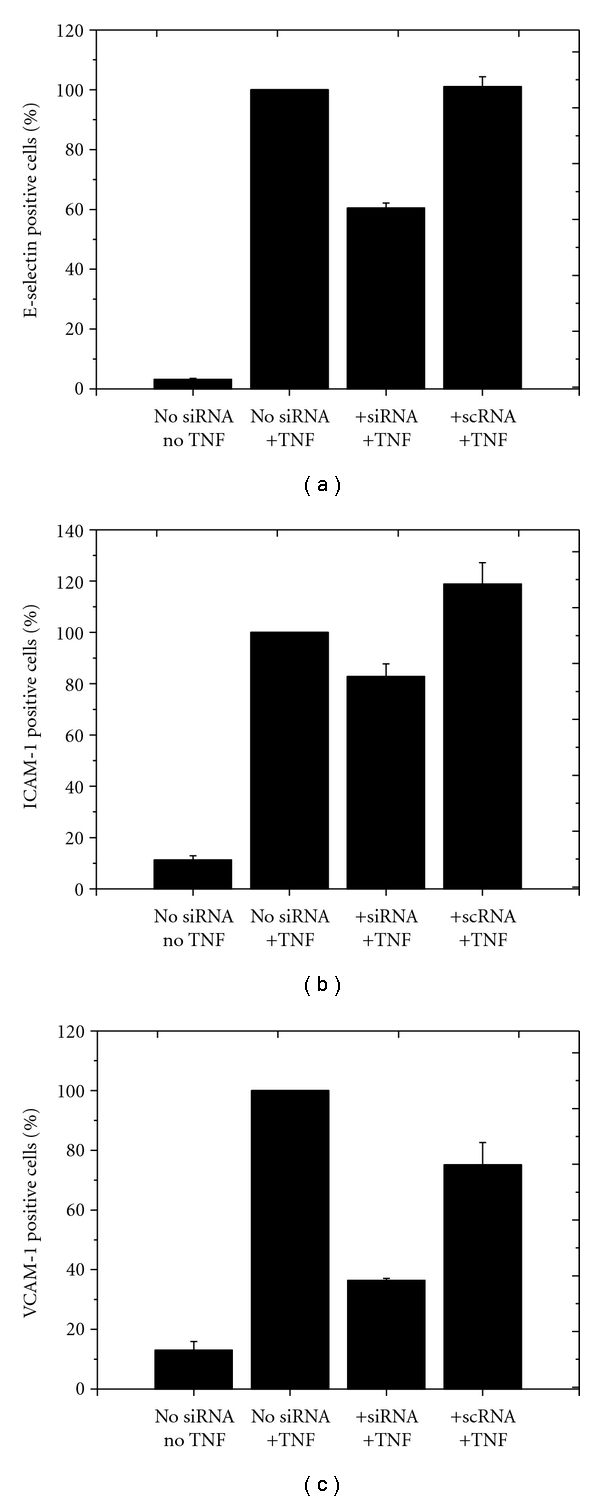
Application of the mixture of 3 different siRNAs targeting ESELE, ICAM-1, and VCAM-1 demonstrated the practicability and efficacy of transfection of multiple siRNAs simultaneously. The *y*-axis reflects the respective adhesion receptor expression (in %), whereby untreated cells stimulated with TNF-*α* (group 2) were set to 100%. *x*-axis: group 1: HLMECs without siRNA transfection and without TNF-*α* stimulation; group 2: HLMECs without siRNA transfection, but with TNF-*α* stimulation; group 3: HLMECs transfected with 3 different receptor specific siRNAs and with TNF-*α* stimulation; group 4: HLMECs transfected with a nonspecific siRNA and with TNF-*α* stimulation. Each bar represents the mean ± SD of *n* = 3 independent experiments.

HLMECs were transfected with the 3 siRNAs in a similar manner as that described in the experiments shown above. However, this was not performed individually but by combining all 3 siRNAs into a cocktail (Figure 3).

#### 2.1.3. qRT-PCR

**Figure 4:**
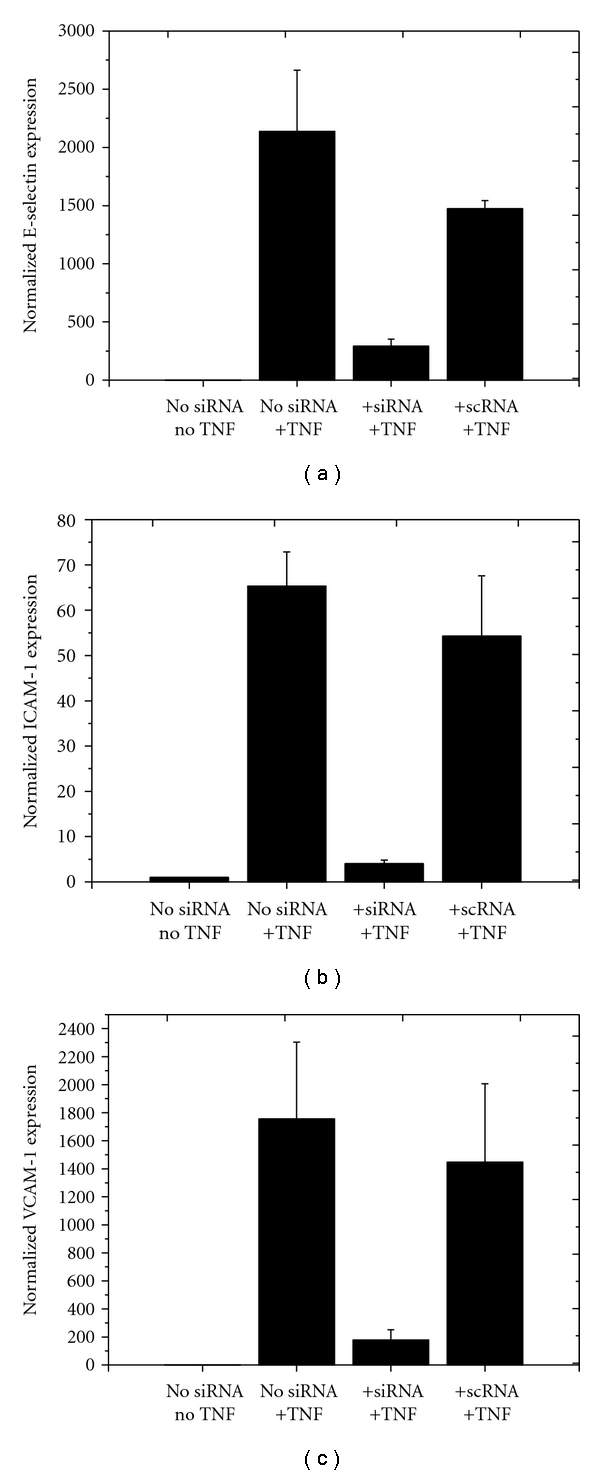
Effects of siRNA and TNF-*α* treatment on ESELE, ICAM-1, and VCAM-1 mRNA levels. Transcript levels were examined by real-time RT-PCR. Each bar represents the mean ± SD of three independent experiments. Gene expression was normalized in relation to group 1, which was set to the value of 1.00. Group 1: HLMECs without siRNA transfection and without TNF-*α* stimulation; group 2: HLMECs without siRNA transfection, but with TNF-*α* stimulation; group 3: HLMECs transfected with a receptor specific siRNA and with TNF-*α* stimulation; group 4: HLMECs transfected with a nonspecific siRNA and with TNF-*α* stimulation.

In addition to the determinations of the protein level of the adhesion receptor expressions, the effects of siRNA transfection on ESELE, ICAM-1 and VCAM-1 mRNA transcript levels were analysed by qRT-PCR (Figures 4(a)–4(c)). Normalized gene expression was calculated by the ΔCt method; GAPDH was used as a reference, and group 1 (nontreated and nonstimulated) was set to the value of 1.00.

The effects of siESELE, siICAM-1, and siVCAM-1 on target protein expression were strongly reflected by changes in the corresponding transcript levels. All three siRNAs (siESELE, siICAM-1 and siVCAM-1) significantly reduced the levels of their corresponding target mRNA compared to the groups treated with TNF-*α* only (*P* < .05). In contrast, the control groups treated with siSCR showed no reduction in respective mRNA levels (Figure 4).

**Figure 5:**
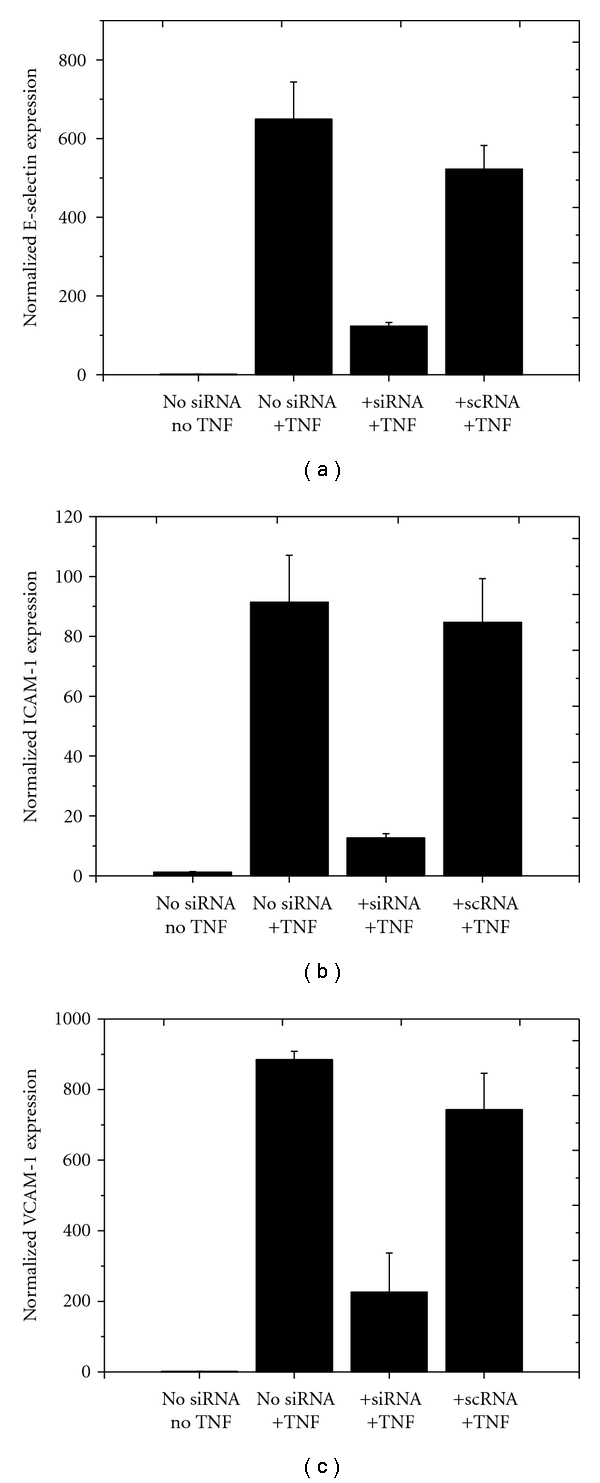
Effects of siRNA and TNF-*α* treatment on ESELE, ICAM-1, and VCAM-1 mRNA levels. Experiments and groups were as described in Figure 4.

Additionally, HLMECs were transfected with the 3 different siRNAs in a similar manner as that described in the experiments shown above. However, this was not performed individually, but by combining all 3 siRNAs into a cocktail (Figure 5). The application of this siRNA cocktail targeting all three different adhesion receptors resulted in a high, significant reduction of all adhesion receptor mRNAs (Figure 5).

#### 2.1.4. Neutrophil Adhesion Assay

**Figure 6:**
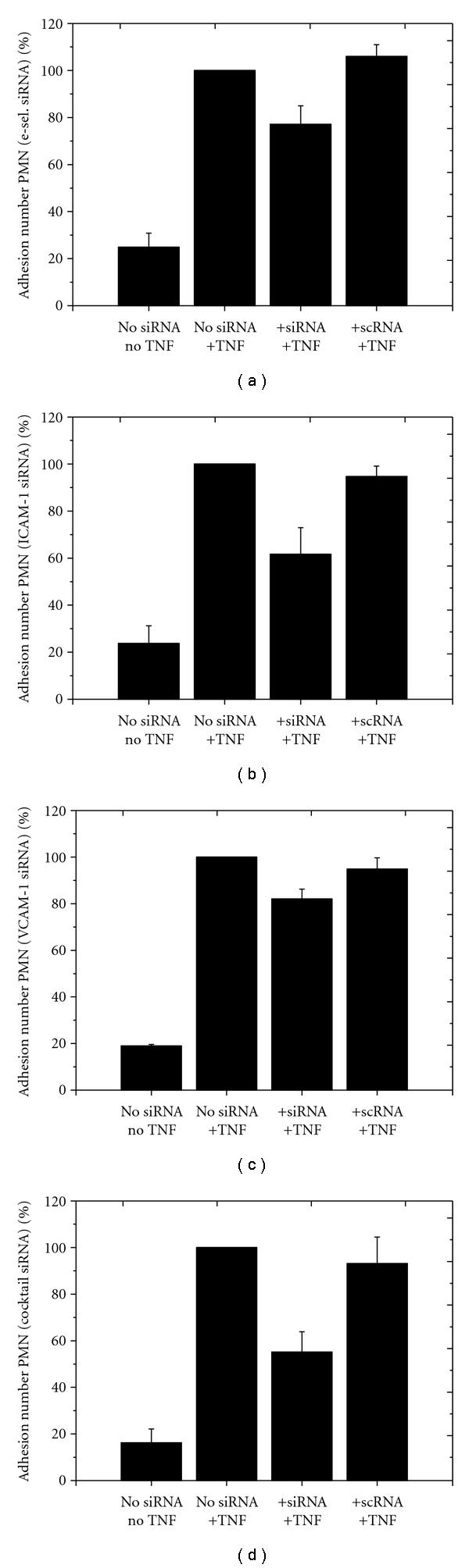
Effects of siRNA and TNF-*α* treatment on binding of neutrophils to confluent layers of HLMECs. The *y*-axis reflects the respective adhesion of neutrophils (in %), whereby untreated cells stimulated with TNF-*α* (group 2) were set to 100%. *x*-axis: group 1: HLMECs without siRNA transfection and without TNF-*α* stimulation; groups 2: HLMECs without siRNA transfection, but with TNF-*α* stimulation; group 3: HLMECs transfected with specific siRNAs targeting ESELE (a), ICAM-1 (b), VCAM-1 (c), or all 3 different siRNAs simultaneously (d) plus TNF-*α* stimulation; group 4: HLMECs transfected with a nonspecific siRNA and with TNF-*α* stimulation. Each bar represents the mean ± SD of *n* = 3 independent experiments, each performed in triplicate.

Two million PMN-neutrophils were added to each well containing confluent layers of HLMECs, and after 1 hour, the adherent neutrophils were quantified using a CASY cell counter system (Figure 6). On the nonactivated cell layer, only approximately 2,000 neutrophils attached. The stimulation of the HLMECs with TNF-*α* led to a more than 4-fold increase (up to 7,000–9,000) in the number of adherent granulocytes (Figures 6(a)–6(d)). Groups transfected with specific siRNA showed significantly reduced adhesion of neutrophils (*P* < .05). Application of siRNA targeting ESELE effected an adhesion of only 5,855 ± 904 PMNs. Transfection with ICAM-1 siRNA (Figure 6(b)) resulted in a diminished adhesion of 4,824 ± 57, while transfection with VCAM-1 siRNA (Figure 6(c)) resulted in an adhesion of 6,375 ± 492. Finally, the cocktail transfection (3 different siRNAs) resulted in 4,325 ± 113 adherent neutrophils (Figure 6(d)).

## 3. Discussion

Lung transplantation carries a higher perioperative morbidity than other transplant procedures [7] including more frequent occurrence of primary graft failure. Among a variety of other findings, histological examinations have shown increased infiltration of leukocytes into the parenchyma, causing damage to the graft and thus reducing graft function [8]. In particular, the vascular endothelium seems to be the graft component that is most vulnerable to the stimulus exerted by ischemia [9]. Endothelial activation leads to expression of adhesion molecules, which, in turn, exert a considerable influence on the interaction between leukocytes and endothelium [10–13]. Basit et al. have demonstrated a correlation between intercellular adhesion molecule 1 (ICAM 1) and recruitment of leukocytes within pulmonary parenchyma [5]. Additionally, in an animal model, Levine et al. have shown that blocking of adhesion molecules exerts a positive influence on reperfusion-associated hyperpermeability of transplanted lungs [14]. These findings indicate that adhesion molecules play an essential role in the modulation of acute as well as chronic graft rejection reactions [15, 16], thus exerting a significant influence on the further development of perioperative morbidity and mortality of transplanted patients. Therefore, suppressing expression of adhesion molecules in pulmonary allografts in order to maintain a high level of graft function seems to be a very promising therapeutic approach. 

To gain a systematic insight into the expression patterns of adhesion molecules on pulmonary microvascular endothelial cells, tumour necrosis factor alpha, a well-known and highly potent cytokine, was used to stimulate expression of adhesion molecules. We have been able to show that various adhesion molecules from the selectin and immunoglobulin families can be stimulated to a significant degree but within different time windows (see Figure 1), which partly differs from the findings published by other investigators [17]. This might be explained by the different cell types used. 

In analogy to the fact that different adhesion molecules exert different functions, we have been able to demonstrate, by means of our experiments, that expression of different adhesion molecules on human lung microvascular endothelial cells reaches a maximum after different latency periods following application of the stimulus. The fact that according to several authors, up to 40% of the pulmonary parenchymal mass might be composed of endothelial cells underlines the particular importance of these findings when it comes to protecting and preserving pulmonary allografts [18].

Furthermore, we were able to show that human lung microvascular endothelial cells can in fact be effectively transfected using a cationic liposomal transfection medium. We decided not to use a viral vector so as to work as closely as possible to actual clinical applicability while at the same time preventing the restriction of our method to a safe laboratory environment. 

In the course of practical application, however, it will not be sufficient to choose a single target or a single adhesion molecule. Instead, it will be necessary to suppress a whole series of adhesion molecules in order to achieve a sustainable effect, and therefore, we have also performed what we call “cocktail transfection.” This means that we have performed transfection of cells with a protective medium containing a combination of the three specific siRNA sequences against ESELE, ICAM, and VCAM and subsequently examined the effect achieved with respect to the individual adhesion molecules. This resulted in a highly significant reduction of expression of the individual adhesion molecules, as shown by FACS analysis, qRT-PCR, as well as by functional assays quantifying neutrophil adhesion. It remains to be shown, of course, whether the results achieved in a laboratory setting under static conditions will be confirmed under dynamic conditions in animal experiments.

The present study has demonstrated that transfection with specific siRNA in each case achieved a significant degree of suppression of the adhesion molecules examined. To the best of our knowledge, so far, this has not been described in the literature on lung microvascular cells. An additional advantage is the transient character of the liposomal transfection method. The silencing is sustained for a period of approximately 2 weeks only ([19] and internal datas), after this period, a normal vascular endothelium showing regular physiological reactions is once again available. 

On the basis of both the results described above and the knowledge of the consequences of pathological interaction between leukocytes and endothelium in transplanted organs, we believe that protecting pulmonary allografts by means of siRNA transfection may represent a promising therapy for improving both short and long-term function of pulmonary grafts.

## 4. Materials and Methods

### 4.1. Primary Cells and Culturing

Human lung microvascular endothelial cells (HLMECs) and the MVECGM medium (microvascular endothelial cell growth medium) were purchased from Sciencell (Carlsbad, Calif, USA). These primary HLMECs are isolated from human lung tissue and are characterized by immunofluorescent methods with antibodies to vWF/Factor VIII and CD31 (P-CAM) and by uptake of DiI-Ac-LDL [20]. HLMECs were cultured at 37°C und 5% CO_2_, according to standard procedures. Subcultures from the third passage were used for all experiments.

### 4.2. Transfection Protocol

One day before siRNA transfection, 150,000 HLMECs per well were cultured in gelatine-coated 12-well plates without antibiotics. After reaching 80% confluence, the cells were transfected with 100 nM specific siRNA targeting E-selectin (ESELE) intercellular adhesion molecule-1 (ICAM-1) or vascular adhesion molecule-1 (VCAM-1) or a nonspecific siRNA (negative control = scrambled siRNA, siSCR) for 2 hours in a serum-free medium, followed by the replacement of the supernatants with ECM without antibiotics. Transfections of siRNA were carried out with cellfectin, 1 ng/ml (Invitrogen, Karlsruhe, Germany). 

Twelve hours later, HLMECs were stimulated with tumour necrosis factor-*α* (TNF-*α*) (Immunotools, Friesoythe, Germany). Keeping in mind the time dependent expression curves of the three adhesion molecules the time span of 12 hours after TNF stimulation ensured a high expression level of all three adhesion molecules. 

Internal not published data showed a dose dependent manner of maximum adhesion molecule expression. For ESELE and ICAM-1, 2.5 ng/mL TNF-*α*, and for VCAM-1, 5 ng/mL TNF-*α* were used as described previously [21–23]. Using these concentrations the specific maximum adhesion molecule expression was not increasable any more.

**Experimental Group Setup**

Negative controls: HLMECs without siRNA transfection and without TNF-*α* stimulation.
Positive controls: HLMECs without siRNA transfection, but with TNF-*α* stimulation.
siRNA groups: HLMECs transfected with a receptor specific siRNA (or a cocktail out of three siRNA) and with TNF-*α* stimulation.
Scrambled-siRNA groups: HLMECs transfected with a nonspecific siRNA and with TNF-*α* stimulation.

### 4.3. siRNA Sequences

Three to five different sequences (each targeting ICAM-1, VCAM-1, or ESELE) were designed by using various Internet-based software tools. The sequences (all synthesized by Qiagen GmbH, Hilden, Germany) showing the highest knockdown were chosen for all further experiments. The following siRNA sequences and concentrations were used for transfection:

ESELE:

sense: 5′-GGUUGAAUGCACCACUCAAdTdT-3′
antisense: 5′-UUGAGUGGUGCAUUCAACCdTdT-3′

ICAM-1:

sense: 5′-GCCUCAGCACGUACCUCUAdTdT-3′
antisense: 5′-UAGAGGUACGUGCUGAGGCdTdT-3′

VCAM-1:

sense: 5′-AAUGCAACUCUCACCUUAAdTdT-3′
antisense: 5′-UUAAGGUGAGAGUUGCAUUdTdT-3′

siSCR (scrambled siRNA):

sense: 5′-UUCUCCGAACGUGUCACGUdTdT-3′
antisense: 5′-ACGUGACACGUUCGGAGAAdTdT-3′

### 4.4. Flow Cytometry (FACS)

Eight hours after stimulation with TNF-*α*, the cells were stained for 1 hour at 4°C with the respective antibodies in 1,000 *μ*L PBS containing 0.5% FCS in a 1 : 300 dilution for ESELE and ICAM-1 and 1 : 200 for VCAM-1. Antibodies (ESELE: PE-Cy5 antihuman CD62E (IgG1 Mouse, clone 68-5H11), ICAM-1: PE antihuman CD54 (IgG1 Mouse, clone HA 58), and VCAM-1: FITC antihuman CD106 (IgG1 Mouse, clone 51-10C9)) were all purchased from BD Biosciences Pharmingen (Heidelberg, Germany). After the staining step, the cells were washed first with 1,000 *μ*L 0.5% FCS in PBS and then with 800 *μ*L HEPES-BBS and detached. The cells were centrifuged at 220 g and fixed with 2.5% paraformaldehyde in PBS. Flow cytometric analyses (5,000 cells/measurement) were performed with a FACScan (Becton Dickinson GmbH) and evaluated with CellQuestPro software (Becton Dickinson GmbH).

### 4.5. Semi-Quantitative Real-Time PCR (qRT-PCR)

Eight hours after stimulation with TNF-*α*, the cells were prepared for qRT-PCR. Total RNA from cultured HLMECs was purified by Aurum total RNA mini kit (Bio-Rad Laboratories, Inc., Hercules, Calif, USA). Consecutively, 200 ng of each RNA sample was reverse transcribed by iScript cDNA Synthesis Kit (Bio-Rad Laboratories, Inc., Hercules, Calif, USA), according to the manufacturer's instructions. All primers were synthesized by Operon Biotechnologies GmbH (Köln, Germany). qRT-PCR was performed as described previously [24]. All qRT-PCRs contained IQ SYBR Green Supermix (Bio-Rad Laboratories, Inc., Hercules, Calif, USA), 400 nM forward and reverse primer and 2 ng of cDNA in a total volume of 15 *μ*L.

**Primer Sequences**

ESELE:

sense: 5′-GCCCAGAGCCTTCAGTGTACC-3′
antisense: 5′-GGAATGGCTGCACCTCACAG-3′

ICAM-1:

sense: 5′-CTTGAGGGCACCTACCTCTGTC-3′
antisense: 5′-CGGCTGCTACCACAGTGATG-3′

VCAM-1:

sense: 5′-ACACTTTATGTCAATGTTGCCCC-3′
antisense: 5′-GAGGCTGTAGCTCCCCGT-3′

All qRT-PCRs were performed in triplicates. Normalized gene expression was calculated by the ΔCt method using GAPDH as a reference.

### 4.6. Maximal Receptor Expression after TNF-*α* Stimulation

Preliminary, not in this paper, demonstrated data showed a dose-dependent fashion of the different adhesion molecules, analogous to already published cell lines. They show a concentration of 2.5 *μ*g TNF for ESELE und ICAM and 5 *μ*g TNF for VCAM to get a expression profile which was not increasable any more. For the siRNA cocktail, we used a TNF concentration of 2.5 *μ*g. One hundred and fifty thousand HLMECs per well were cultured and stimulated with TNF-*α*. Every 2 hours for the next 24 (or 48) hours, the expression of ICAM-1, VCAM-1, or ESELE was measured by FACS.

### 4.7. Adhesion of Neutrophils to Activated HLMECs

Iso-osmotic Percoll (GE Healthcare Bio-Science AB, Uppsala, Sweden) was formed by adding 0.18 g NaCl to 20 mL Percoll. The Percoll solution was then diluted with 0.15 M NaCl to final concentrations of 55% and 74%; 3 mL of each solution was used to form the gradient. Human blood was diluted 1 : 1 with PBS, and 3 mL of this solution was placed on the top of each gradient. The gradients were centrifuged at 350 g for 20 minutes; cells were collected from the interface and washed with 6 mL of EGM-2. Erythrocytes were lysed by incubating the cells with 5 mL of lysing buffer (8.29 g/L ammonium chloride, 1 g/L potassium hydrogen carbonate, 0.037 g/L Na_2_-EDTA-2H_2_O). After stimulating the endothelial cells with TNF-*α*, the medium was removed and 2 million granulocytes in 1 mL EGM-2 were added to each well. After 1 hour, the granulocyte suspension was gently removed, and the wells were washed with medium twice. The adherent granulocytes were detached (Detachment Kit, Promocell GmbH, Heidelberg, Germany), resuspended in 100 *μ*L medium and quantified using a CASY cell counter system (Schärfe System, Reutlingen, Germany).

### 4.8. Statistics

The results are expressed as mean ± standard deviation (SD). The results represent the average of three independent experiments (each performed in triplicate). Adhesion assay: The number of attached neutrophils on TNF-*α* stimulated HLMECs was adjusted to 100% (positive control), and the number of attached cells from the experiments with specific or scrambled siRNA was calculated in relation to the 100% of the control. 

Statistical analyses were performed using the statistics software package SPSS (SPSS Software Inc., Chicago, Ill, USA). Differences between the groups were calculated by *F*-test followed by a Post hoc test (Tukey HSD). We considered values of *P* < .05 significant.

## Abbreviations

ESELE: E-Selectin
FACS: Fluorescence-activated cell sorting
GAPDH: Glyceraldehyde-3-phosphate dehydrogenase
HLMVEC: Human lung microvascular endothelial cells
ICAM: Intercellular adhesion molecule
mRNA: Messenger RNA
PGF: Primary graft failure
PMN-neutrophils: Polymorphonuclear neutrophils
qRT-PCR: Real-time quantitative reverse transcription
RISC: RNA-induced silencing complex
RNA: Ribonucleic acid
siSCR: Small interfering scrambled
siRNA: Small interfering RNA
TNF: Tumour necrosis factor
VCAM: Vascular adhesion molecule.
